# Prevalence and Predictors of Cisplatin-Induced Peripheral Neuropathy at the Kenyatta National Hospital

**DOI:** 10.1200/JGO.19.00097

**Published:** 2019-09-03

**Authors:** Mohammed S. Ezzi, Nicholas A. Othieno-Abinya, Erastus Amayo, Peter Oyiro, Angela McLigeyo, Robert Bett Yatich, Bonginkosi Shoba

**Affiliations:** ^1^ University of Nairobi, Nairobi, Kenya; ^2^ Moi Teaching and Referral Hospital, Eldoret, Kenya; ^3^ University of KwaZulu-Natal, KwaZulu-Natal, South Africa

## Abstract

**PURPOSE:**

To determine the prevalence, predictors, and/or risk factors of chemotherapy-induced peripheral neuropathy in patients undergoing chemotherapy with cisplatin at Kenyatta National Hospital, Nairobi, Kenya.

**METHODS:**

This was a cross-sectional analysis of patients who underwent chemotherapy with cisplatin for at least 2 months at Kenyatta National Hospital oncology units. Peripheral neuropathy was determined by history and physical examination per the protocol. Data are presented in tables. Descriptive inferential statistics such as means, medians, and proportions were determined where applicable.

**RESULTS:**

We recruited 67 patients who were undergoing chemotherapy with cisplatin. Fifty-six patients (83.6%) had peripheral neuropathy. Forty-five patients (81%) had mild-grade (grades 1 and 2) peripheral neuropathy. Only two patients (3.1%) had grade 4 neuropathy. Almost all patients who were overweight or obese developed peripheral neuropathy.

**CONCLUSION:**

Peripheral neuropathy among patients receiving cisplatin is quite prevalent at Kenyatta National Hospital (83.6% prevalence rate). However, most of the patients had a mild grade of neuropathy, which is largely consistent with literature elsewhere.

## INTRODUCTION

Chemotherapy-induced peripheral neuropathy (CIPN) is a major adverse effect of various chemotherapeutic drugs for cancer.^1^ Platinum agents, especially cisplatin, are an important group of chemotherapeutic drugs. They are used in the management of various cancers with curative and/or palliative intent. However, these drugs are associated with numerous adverse effects. Peripheral neuropathy is a major nonhematologic adverse effect associated with platinum agents.^2^

The prevalence of CIPN is high. Seretny et al^3^ reported a prevalence rate of 68%. Moreover, the prevalence rate of CIPN was still 30% after 6 months of chemotherapy. CIPN may develop during chemotherapy or several months after therapy, and symptoms may persist for a longer duration of time post treatment.^4^

Clinical symptoms of CIPN mostly involve the peripheral nervous system. These may manifest as sensory loss, paresthesia, numbness, or tingling sensation and pain in a “stocking and glove” distribution.^1^ CIPN and related symptoms have a significant negative impact on a patient’s quality of life. CIPN is also associated with other comorbidities^5^ and a heavy economic cost.^6^

Currently, there are neither preventive measures nor curative treatments for CIPN.^7^ Developing CIPN may lead to either dose reduction or discontinuation of chemotherapy. This may have an adverse impact on the patient’s outcome. Although CIPN is a significant complication of cisplatin chemotherapy, there are no data on its overall impact, prevalence, and risk factors in our setting of Nairobi, Kenya. Therefore, to the best of our knowledge, this study is the index study that has highlighted the magnitude of CIPN in our setting. We evaluated the prevalence and severity of peripheral neuropathy among patients receiving cisplatin-based chemotherapy. We also examined the factors associated with peripheral neuropathy.

## METHODS

This cross-sectional study was conducted for a period of 4 months, from April to July 2018. All patients 13 years or older, who were undergoing or had received cisplatin-based chemotherapy for at least 2 months at the oncology unit of Kenyatta National Hospital, were eligible for inclusion in the study. Kenyatta National Hospital is a national teaching and referral hospital in Nairobi, Kenya. Patients were recruited during their scheduled appointments. Informed consent or assent was obtained from all patients before inclusion into the study. The study was approved by the Kenyatta National Hospital/University of Nairobi Ethical Committee (P63/02/2018).

**CONTEXT**

**Key Objective**
Peripheral neuropathy is a common and significant chronic complication of platinum agents such as cisplatin. The prevalence of this complication is not known in the Nairobi region of Kenya.
**Knowledge Generated**
Health workers should be trained to screen, evaluate, and manage peripheral neuropathy in the initial stages of chemotherapy with cisplatin.
**Relevance**
The current study sought to determine the prevalence of peripheral neuropathy as a result of cisplatin therapy and its risk factors at Kenyatta National Hospital in Nairobi, Kenya.

Demographic and clinical data were collected using questionnaires with closed and open-ended questions. A focused neurologic examination was performed per Total Neuropathy Score (TNS) requirements.^8^ The presence of neuropathy was graded accordingly. Chart review was performed to extract baseline laboratory blood values such as hemoglobin, albumin, and creatinine levels. Demographic and clinical parameters documented include age, sex, type and stage of cancer, cumulative dose, concurrent other neurotoxic chemotherapy drugs or radiotherapy, hypoalbuminemia (serum albumin levels less than 35.0 g/L), anemia (hemoglobin levels less than 12.0 g/dL in women and less than 13.0 g/dL in men), diabetes mellitus, chronic renal failure (glomerular filtration rate less than 60.0 mL/min/1.73 m^2^), HIV infection, habit of alcohol consumption, and smoking history.

Data were entered and managed in a Microsoft Excel 2013 spreadsheet. Statistical analysis was performed using SPSS version 21.0. The dependent variable was the prevalence of CIPN; independent variables were predictors or risk factors for CIPN.

The study population was described by summarizing demographic data and clinical characteristics in percentages and means or medians for categorical and continuous variables, respectively. The prevalence of CIPN was presented as a percentage with 95% CI. The relationship between CIPN and selected demographic data and clinical characteristics was used to determine the risk of developing CIPN. These associations were determined using the χ^2^ test, and the comparison of means was done using an independent *t* test. Odds ratios were calculated and presented as the relative risk associated with CIPN.

## RESULTS

### Clinical Characteristics of Patients

Among 67 participants who were undergoing cisplatin therapy, the median age was 51 years (range, 14 to 80 years) with a male to female ratio of 1:1.7. The most common tumor sites were in the genitourinary region, which accounted for 40% of all tumor sites. Genitourinary site tumors included those from the ovary, uterine cervix, corpus uterus, testis, and urinary bladder. Most patients had stage III (41.8%) and stage IV (44.8%) disease. Table 1 provides a detailed summary of patient characteristics.

**TABLE 1:**
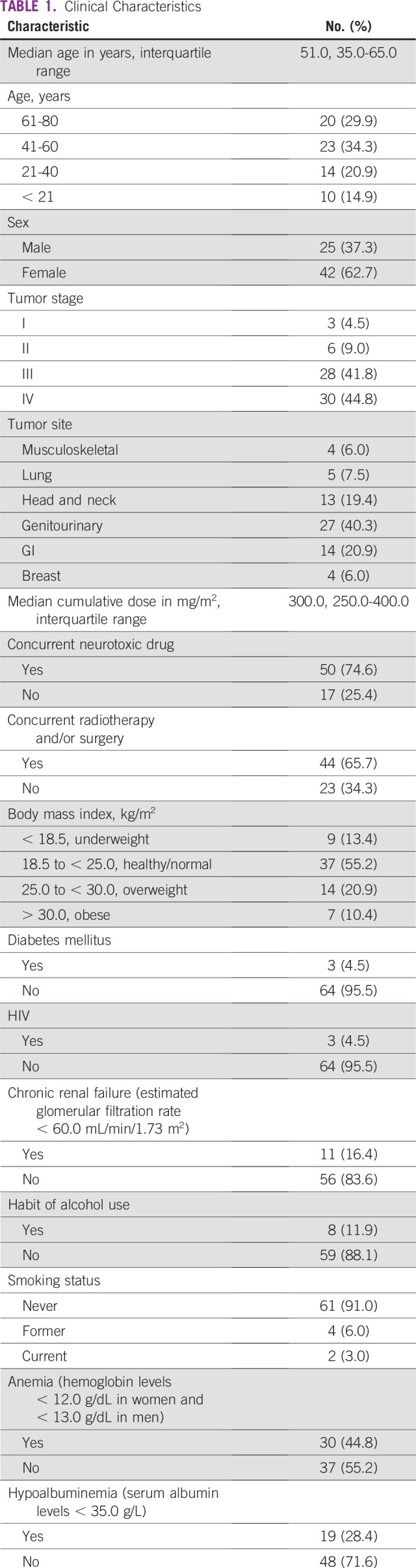
Clinical Characteristics

### Prevalence of Peripheral Neuropathy

At the time of the study, 56 patients (83.6%) had neuropathy (TNS greater than 2), of whom 45 patients (81%) had mild neuropathy. Two patients had grade 4 neuropathy (Table 2).

**TABLE 2:**
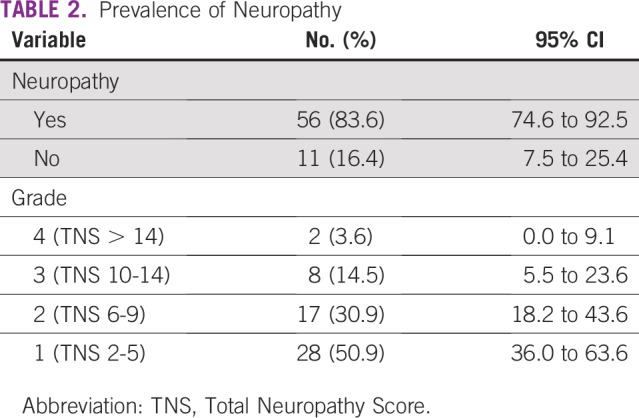
Prevalence of Neuropathy

### Risk Factors and Development of Neuropathy

Thirty-seven patients (55%) had a normal body mass index. Seven patients (10%) were obese (body mass index greater than 30 kg/m^2^). Almost all patients who were overweight or obese developed peripheral neuropathy, but this was not statistically significant (*P* = .999 and *P* = .704, respectively).

Fifty patients (74.6%) were receiving cisplatin combined with another neurotoxic chemotherapeutic agent. Forty-one patients (82%) who were receiving a concurrent neurotoxic chemotherapeutic agent developed peripheral neuropathy; however, this was not statistically significant (*P* = .716). The most frequently combined neurotoxic chemotherapeutic drug was taxane (44 patients were receiving taxane). Patients who were receiving combined cisplatin and taxane had a higher median TNS of 5 (range, TNS 0 to 17) versus those without taxane combined with cisplatin (median TNS, 3; range, TNS 0 to 14). The association between the development of neuropathy and risk factors evaluated is shown in Table 3.

**TABLE 3:**
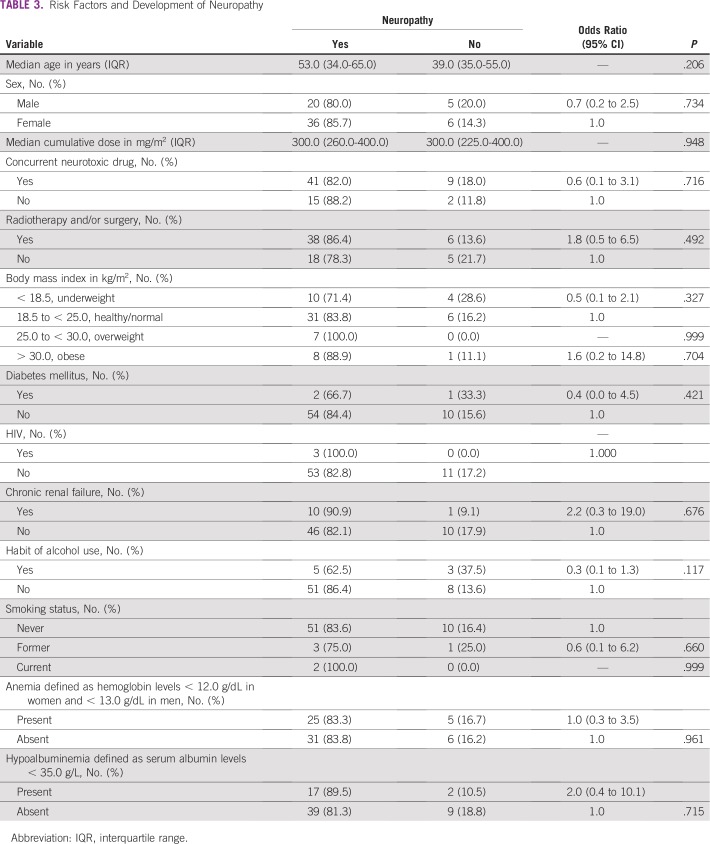
Risk Factors and Development of Neuropathy

## DISCUSSION

To our knowledge, this is the first cross-sectional study on the prevalence of CIPN in Sub Sahara Africa. It has provided a profile of CIPN in patients treated with cisplatin. The median age of the study population was 51 years, which is a relatively young population, and most of the patients had advanced disease. This is comparable with the local cancer registry, which states that 60% of Kenyans affected with cancer are younger than 70 years and that 70% to 80% of cancers are diagnosed at late stages.^9^

Our study found the prevalence rate of CIPN to be 83.6%, of which most patients had mild to moderate peripheral neuropathy. The prevalence rate of CIPN ranges from 20% to 90%.^10-17^ This wide range may be influenced by several factors such as study population, length of follow-up, chemotherapy regimen, and assessment tools used.

A cross-sectional study of 29 patients by Vasquez et al^18^ found that all patients had clinical evidence of neuropathy by the fourth cycle of chemotherapy. In addition, Kandula et al^19^ reported a prevalence rate of 83% among patients who had finished chemotherapy. A meta-analysis by Seretny et al^3^ found the overall prevalence rate of CIPN to be 48%. However, these authors noted that two large studies evaluating cisplatin neurotoxicity did not evaluate for mild grades of neuropathy and could have lowered the prevalence rate.^14,20^

Not much information is available on demographic data and clinical characteristics that increases the risk for CIPN development. In most registry studies, risk factors for CIPN were neither evaluated nor demonstrated to influence the prevalence and severity of CIPN.^21-23^ However, a study by the Southwest Oncology Group identified older age, prior or concurrent treatment with a neurotoxic drug, and decreased creatinine clearance as risk factors for the development of CIPN.^24^

This study has several limitations. The cross-sectional design assesses clinical features of peripheral neuropathy at one point in time. It cannot assess nor determine the progression and evolution of clinical features. Furthermore, we were not able to assess the impact of CIPN on quality of life and therapies used to mitigate peripheral neuropathy. The associations identified between demographic data and clinical characteristics warrant confirmation as determinants of CIPN in a prospective study. In addition, data were obtained from a single tertiary hospital, hence introducing a potential for selection bias. The study only collected data from patients who had received or were receiving cisplatin-based chemotherapy. Hence the findings of this study cannot be generalized to patients receiving other neurotoxic chemotherapy drugs.

In conclusion, we found that the development of peripheral neuropathy as a result of cisplatin-based therapy is quite prevalent (83.6% prevalence rate). Most of our patients had mild peripheral neuropathy. Long-term prospective studies are needed to determine the natural course, associated risk factors, and attenuating factors for CIPN.
